# Fecal sample collection methods and time of day impact microbiome composition and short chain fatty acid concentrations

**DOI:** 10.1038/s41598-021-93031-z

**Published:** 2021-07-07

**Authors:** Jacquelyn Jones, Stacey N Reinke, Alishum Ali, Debra J Palmer, Claus T. Christophersen

**Affiliations:** 1 Trace and Environmental DNA Laboratory, School of Molecular and Life Sciences, Curtin University, Bentley, WA Australia; 2 The Western Australian Human Microbiome Collaboration Centre, Curtin University, Bentley, WA Australia; 3 Centre for Integrative Metabolomics and Computational Biology, School of Science, Edith Cowan University, Joondalup, WA Australia; 4 Telethon Kids Institute, University of Western Australia, Nedlands, WA Australia; 5 School of Medicine, University of Western Australia, Crawley, WA Australia; 6 School of Medical & Health Sciences, Edith Cowan University, Joondalup, WA Australia

**Keywords:** Microbial communities, Sequencing

## Abstract

Associations between the human gut microbiome and health outcomes continues to be of great interest, although fecal sample collection methods which impact microbiome studies are sometimes neglected. Here, we expand on previous work in sample optimization, to promote high quality microbiome data. To compare fecal sample collection methods, amplicons from the bacterial 16S rRNA gene (V4) and fungal (ITS2) region, as well as short chain fatty acid (SCFA) concentrations were determined in fecal material over three timepoints. We demonstrated that spot sampling of stool results in variable detection of some microbial members, and inconsistent levels of SCFA; therefore, sample homogenization prior to subsequent analysis or subsampling is recommended. We also identify a trend in microbial and metabolite composition that shifts over two consecutive stool collections less than 25 h apart. Lastly, we show significant differences in bacterial composition that result from collecting stool samples in OMNIgene·Gut tube (DNA Genotec) or Stool Nucleic Acid Collection and Preservation Tube (NORGEN) compared to immediate freezing. To assist with planning fecal sample collection and storage procedures for microbiome investigations with multiple analyses, we recommend participants to collect the first full bowel movement of the day and freeze the sample immediately after collection.

## Introduction

Our understanding of the relationship between the human gut microbiome and host continues to expand from explorations which describe inhabitants, to studies which demonstrate the involvement of the microbiome in human health and disease and disorders. Some examples include neurological disorders such as depression^1^, Alzheimer’s disease^2^ and Autism Spectrum Disorder^3^, as well as inflammatory diseases such as food allergies^4^, and inflammatory bowel diseases^5^. Advancements in microbiome studies have been accelerated by increased sequencing capabilities^6^, along with sensitive analytical techniques tailored for the quantification of metabolites in fecal material^7,8^. Short chain fatty acids (SCFA) are metabolites produced exclusively by resident bacteria, and are important for proper gut barrier functioning. Therefore, SCFA are also associated with dysbiosis, and other inflammatory disorders^5^; and investigating the gut microbiome by combining microbial sequencing data and metabolomic approaches has been an important step in unraveling links between resident bacteria, SCFA, and health outcomes^9–11^. However, stool, which is used as proxy for the distal colon microbiome, is a complex matrix of endo- and exogenous material containing a heterogeneous distribution of microorganisms^12^, which is susceptible to changes during and after collection.

Microbiome profiles may be misrepresented due to subsampling of non-homogenized stool as it has previously been shown that large variations in bacterial abundance detected via qPCR in non-homogenized stool samples were significantly reduced after stool homogenization^13^. In addition, the effects of sub-sampling stool may be further amplified when performing metabolomic analyses, as highly sensitive techniques are used^8,14^. Lastly, microbiome profiles can also be influenced by sequencing depth, as shown by random subsampling of shotgun metagenomic sequence data^15^.

Stool collection by participants may be an undesirable yet necessary aspect of partaking in a microbiome study. Providing participants with a clean and simple collection method should increase compliance, but also maintain sample integrity. Some commercial stool collection tubes allow for easy collection and short term (~ 14 days) ambient temperature storage; however, some of these have been associated with changes in proportions of bacterial phyla^16^. A final consideration is the level of inter-individual differences that occur in the fecal microbiome over a week^17^, and even from day to day^18^, meaning that collection periods may need to span a number of days, or be collected at a particular time in the day to accurately capture the inherent variability. As far as the authors are aware spatial and short-term temporal variability of bacterial and fungal communities has never been evaluated together with SCFA composition. To address this gap, this study will assess the effects of five fecal sample collection methods, as well as consecutively collected whole stool samples (less than 25 h apart), on the variability of the fecal microbiome. The comparisons will be drawn from bacterial and fungal community composition as well as SCFA profiling.

## Results

### Overview of microbiome taxonomy and SCFA concentrations

Stool samples yielded bacterial communities (bacteriome) from all individuals and sampling methods, while fungal communities (mycobiome) were successfully sequenced in 53 of 78 samples, but with uneven library size (~ 100×). Overall, the fecal bacteriome had a higher number of ASVs than the mycobiome (Supplementary Table 1). Across all individuals and collection methods, the most abundant bacterial families were Bacteroidaceae and Lachnospiraceae, which made up 38% and 10% of the bacteriome, while the most abundant fungal families were Saccharomycetaceae 90%, and Phaffomycetaceae 7%. To account for technical bias in library preparation, a single sample from one individual was also processed in duplicate. Bacterial α-diversity estimates for this replicate sample were more similar than the fungal replicate, while fungal replicates also had low richness, indicating that both the rarity of this community, and the library preparation may impact the interpretation of relative abundance of fungal communities (Supplementary Table 1).

Bacterial and fungal mock communities were also sequenced as positive controls, which allowed reads of the mock community samples (positive control) to be compared to the known composition of the mock community (Supplementary Fig. 1). Still, both mock communities contain a number of species that are not expected to be captured in the human fecal microbiome, and therefore, may not be amplified by the selected primers. Of the 20 bacterial species known to be in the mock community, 18 were correctly detected to family level and 15 to genus level, leaving two species of the mock community unidentified. At the level of order, the proportion of each ASV in the positive control was compared to the known percentage contribution of the mock community. Actinomycetales, Campylobacterales and Rhodobacterales were under represented at 3.5, 3.2, and 4.6 times less that what was expected; while Bacillales and Clostridiales were over represented at 3.4 and 4.4 times more than expected. Of the 19 fungal species in the mock community 13 were sequenced and correctly resolved to family and genus (however, *R. irregularis* only had 11 reads), leaving six species of the mock community unidentified. However, four of these species (*Chytriomyces hyalinus*, *Rhizomucor miehei*, *Rhizoctonia solani*, and *Ustilago maydis*) are not expected to be part of the human microbiome, and were not detected with the ITS2 primers developed for use in the human fecal microbiome.

**Figure 1:**
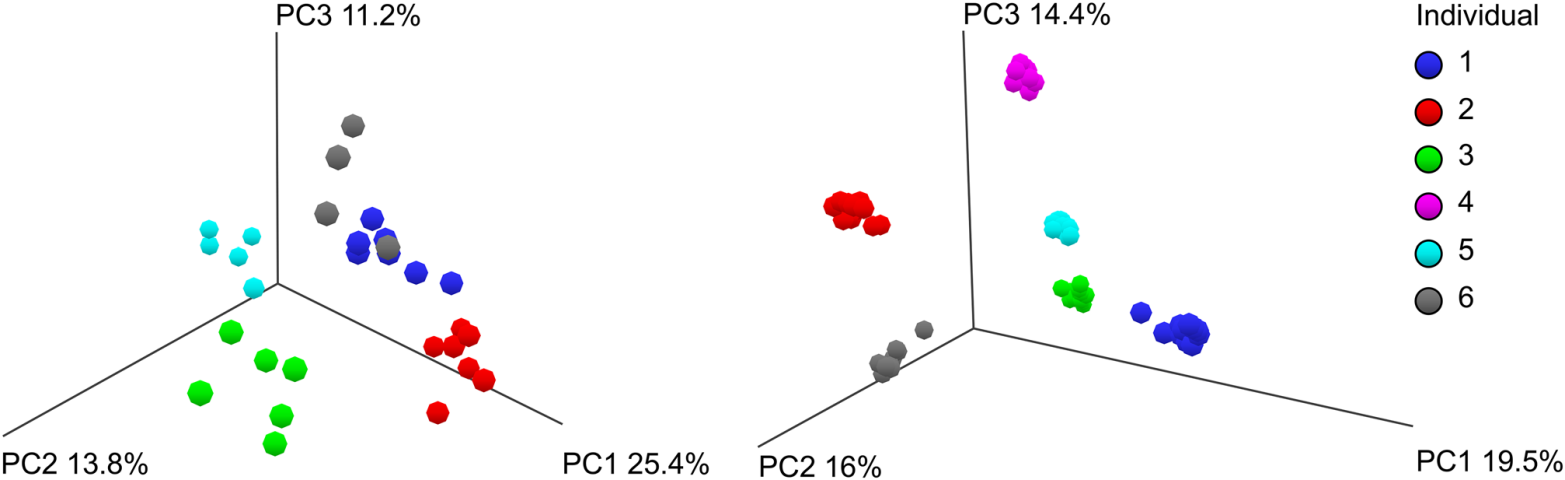
Clustering of microbiome communities per individual from all directly frozen stool samples collected per individual. Plots show the mycobiome (**A**) of each individual is less distinct than bacteriome (**B**). Data was CLR transformed and ordination based on Euclidian distances.

The mycobiome signature of each subject was not as distinct as the bacteriome (Fig. 1). While fungal communities tended to cluster by subject, an analysis by PCoA, shows the mycobiome of individuals one and six overlaps. This seems to be driven in both individuals by Saccharomyces dominating the composition by ≥ 99%. Further, when a distance based (Euclidian, and Bray–Curtis) ordination β-diversity analysis was conducted on abundance data between individuals over the collection period—with all sample types and sample points—significant differences in both bacterial and fungal communities were detected (PERMANOVA p < 0.02).

SCFA concentrations were determined from whole stool as well as surface collected aliquots, and overall the average molar ratio of acetate, propionate, and butyrate was 78:12:10 respectively. The mean concentrations of individual or total SCFA in µmol per gram of feces was not significantly different between collection methods; and in all subjects, acetic acid was most variable, ranging from an average of 103–697 µmol g^−1^.

### Comparison of surface aliquots and whole stool sampling methods

**Figure 2:**
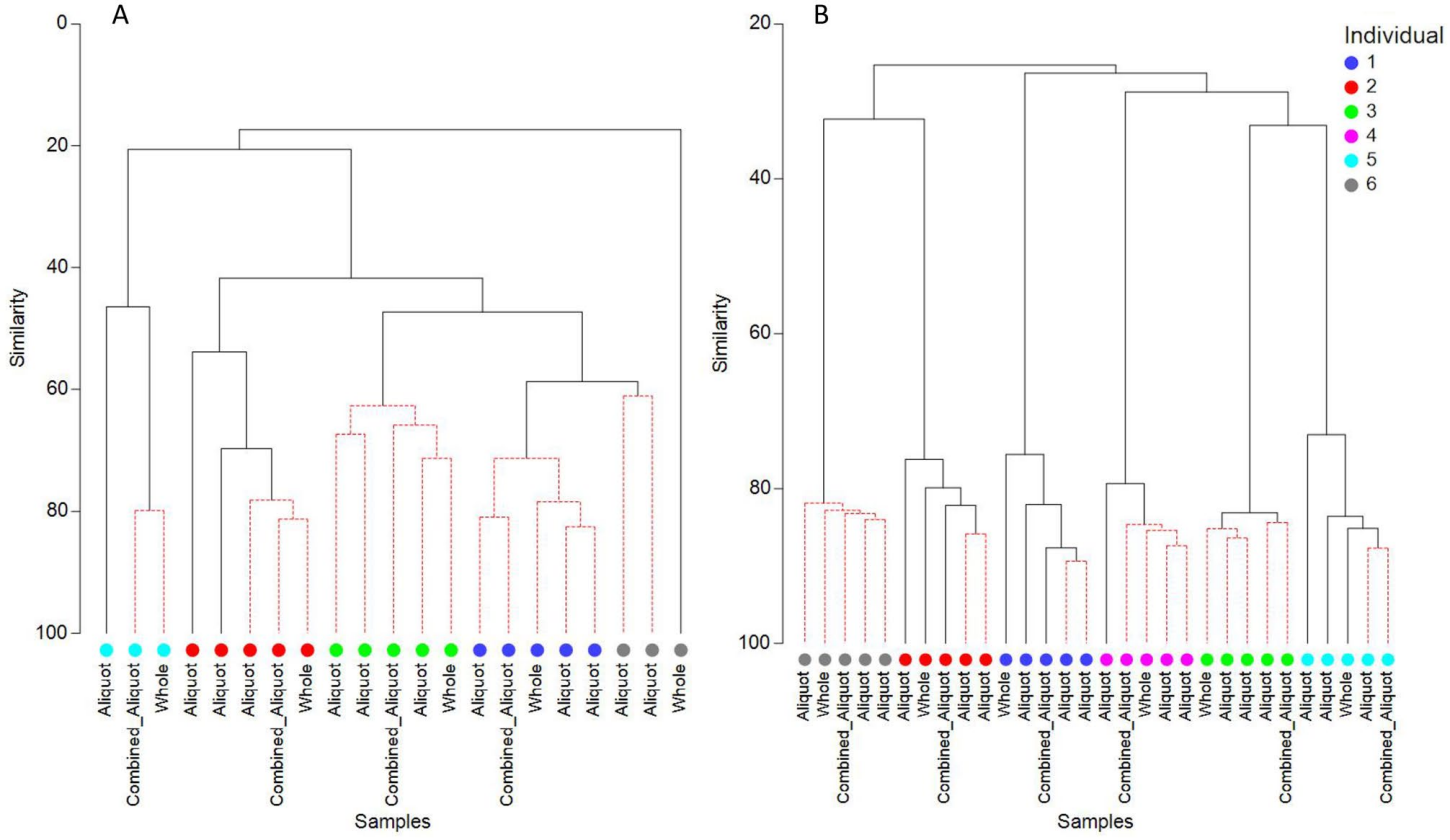
Clustering mycobiome (**A**), and bacteriome (**B**) from whole stool, aliquot, and combined aliquot sampling methods. A SIMPROF test with alpha = 0.05 was used to determine significantly similar sub groupings within individuals, with red dashed lines grouping samples which are significantly similar, and solid black likes grouping samples which are not significantly similar. Group average cluster analysis (9999 repeats) on Bray–Curtis similarity.

To assess the impact of sampling method on a-priori grouping by individual, a hierarchical cluster analysis was performed on bacterial and fungal communities (Fig. 2). Bacterial communities from the same subject grouped together, with a SIMPROF test identifying significantly different sub clusters for five of six individuals. Fungal communities also clustered according to individual, but these groups were less similar than their respective bacterial groups. Furthermore, the aliquots from participant six clustered more closely to participant one than to its own respective whole stool sample. The mycobiome and bacteriome from the combined aliquot clustered according to individual, however did not seem to align consistently with the other aliquots.

**Figure 3:**
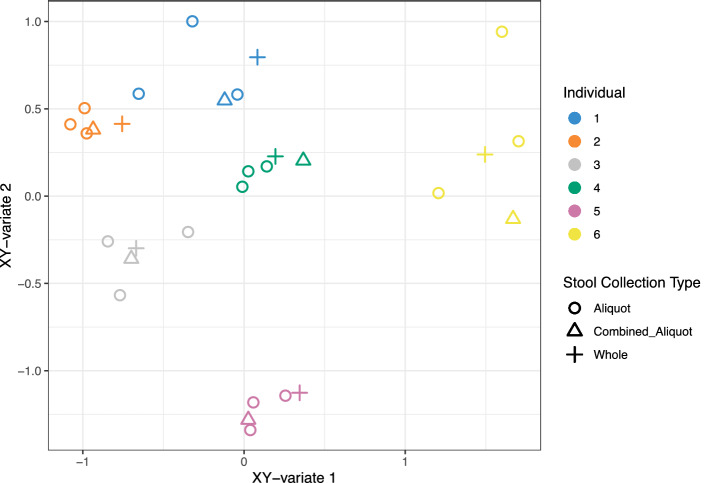
Variability of bacterial community and SCFA composition due to stool homogenization methods. Datasets where combined using rCCA in the R package MixOmics^61^, correlation coefficients were plotted in the shared X–Y space. Bacterial ASV counts were CLR transformed, and SCFA concentration were log10 transformed.

To assess the heterogeneity of SCFAs and bacterial diversity within a single stool, the coefficient of variation (CV) for these measures was compared across three aliquots from a single stool and three separate stools (collected over 3 days) per individual (Supplementary Table 2). Acetic acid and valeric acid, were found to be as variable along a single stool as they were across three bowel movements, whereas propionic was more variable across bowel movements. Overall, the SCFA concentrations were more variable across three bowel movements than along a single stool except in individuals one and six. Shannon diversity was less variable along a single stool (five of six individuals), Chao1 species richness was more variable along a single stool (four of six individuals), and Phylogenetic diversity was equally variable along a single stool (three of three individuals). This trend was further assessed by integrating SCFA data with bacteriome data through rCCA (distance between features were relatively short, indicating the strong agreement between datasets), and the plotted canonical variates show the variability between the surface collected samples was still evident in subjects 1, and 6 (Fig. 3).

Within each individual the composition of microorganisms within the aliquots were not identical to each other, or to the whole stool from which they were sub sampled. DESEq2 was used to compare differentially abundant ASVs between aliquots and whole stool. This method identified 12 bacterial, and 16 fungal features with a log_2_ fold change greater than |2.5|. Of these, five bacterial and one fungal ASVs were significantly enriched in the whole stool compared to aliquots, and one fungal ASV was enriched in stool aliquots compared to whole stool (Table 1).

**Table 1:** ASVs identified with log2 fold change in gematric mean abundance greater than |2.5| between homogenized whole stool and stool aliquots.

ASV	Taxa	log2 fold change
**Bacteria**		
591	*Anaerotruncus massiliensis*	6.62
790	Anaerovoracaceae	17.92^a^
802	Anaerovoracaceae	17.92^a^
70	*Eubacterium* sp.	2.89
4	*Fecalibacterium prausnitzii*	− 3.41
27	*Fecalibacterium prausnitzii*	2.71
405	*Fecalibacterium prausnitzii*	17.47^a^
717	*Oscillibacter ruminantium*	19.52^a^
461	Rhizobiaceae	18.36^a^
**Fungi**		
19	*Alternaria alternata*	− 2.9
219	*Aspergillus niger*	3.9
119	*Aureobasidium pullulans*	17.2^a^
8	*Cyberlindnera jadinii*	3.9
4	*Eremothecium sinecaudum*	− 3.5
41	*Hanseniaspora uvarum*	− 24.1
11	*Hanseniaspora uvarum*	− 8.6
5	*Kazachstania barnettii*	− 22.5
2	*Kazachstania servazzii*	− 21.4^a^
132	*Rhodotorula mucilaginosa*	9.1
29	*Saccharomyces cerevisiae*	− 23.5
6	*Saccharomyces cerevisiae*	4.9
17	*Sporopachydermia lactativora*	5.0
40	*Wickerhamomyces ciferrii*	7.3

^a^Significant enrichment.

### Bacterial community composition is affected by collection methods

**Figure 4:**
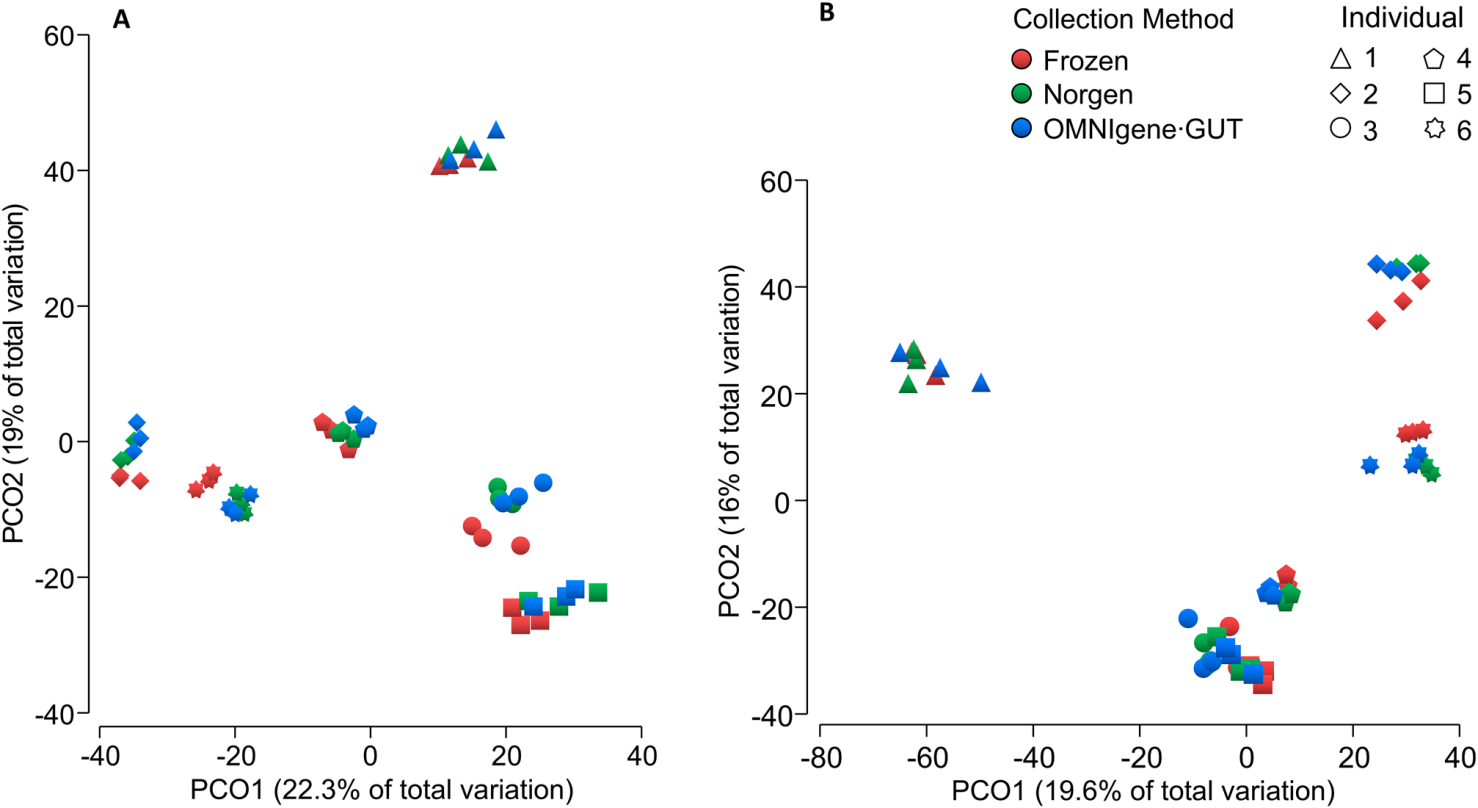
Principal coordinates analysis of β-diversity showing stool samples collected in frozen (n = 18), Norgen (n = 18) and OMNIgene·Gut (n = 18) tube types. Clustering shows directly frozen samples are easily distinguished from stool collected in stabilizing liquid. Data shown in (**A**) was 4th root transformed, using Bray–Curtis similarity distance and (**B**) CLR transformed and Euclidian distance.

**Figure 5:**
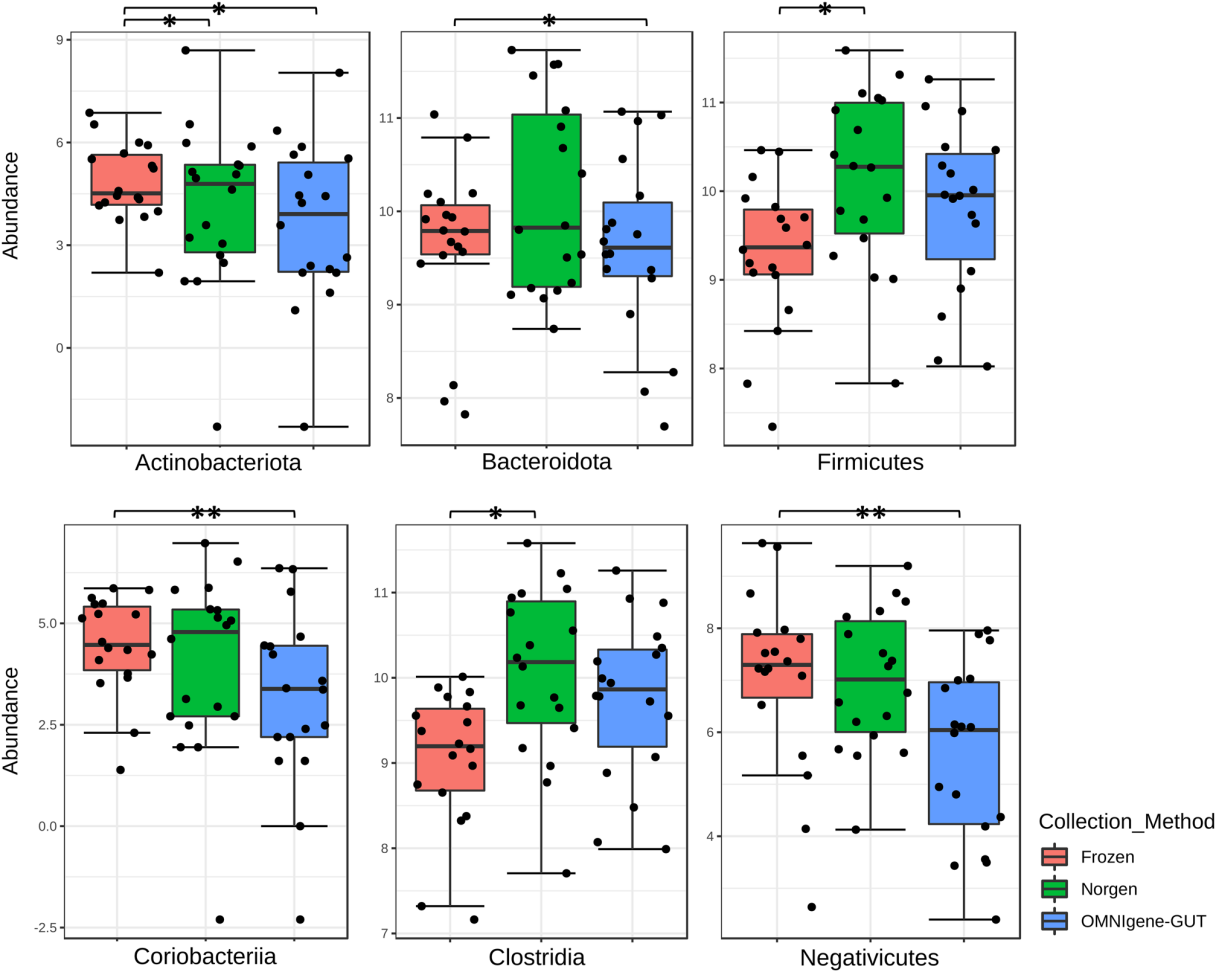
Bacterial phyla with significantly different abundance due to collection method. Groups identified by * are significantly different prior to FDR correction and ** after FDR correction.

Differences in bacterial communities due to collection method were visualized using PCoA (Fig. 4). The ordination plots showed a clear separation between directly frozen samples (method F) and those collected with either the Norgen (collection method N), or OMNIgene·Gut tubes (collection method O). This separation was confirmed to be significant after β-diversity (both Bray–Curtis and Euclidian distances) analysis using PERMANOVA, and stool collected with method F was significantly different (p < 0.01 for Bray–Curtis and Euclidian distance) to both those collected with the N or O methods. Furthermore, the N and O method where also significantly different to each other (p < 0.01 for Bray–Curtis; and p = 0.04 for Euclidian distance). Relative taxonomic abundance analysis of whole stool samples according to collection methods revealed that overall the most abundant families were Bacteroidaceae (F 38%, N 43%, O 39%), Ruminococcaceae (F 7%, N 17%, O 33%), and Lachnospiraceae (F 10%, N 15%, O 10%), with the abundance of Ruminococcaceae significantly increased (ANOVA p < 0.001, FDR = 0.007) due to collection using the N and O methods compared to the F method. A number of taxa were also recovered differentially between the three collection methods (Supplementary Table 3), including some high-ranking taxa (Fig. 5).

### Short term changes to Microbiome composition and SCFA concentration

**Figure 6:**
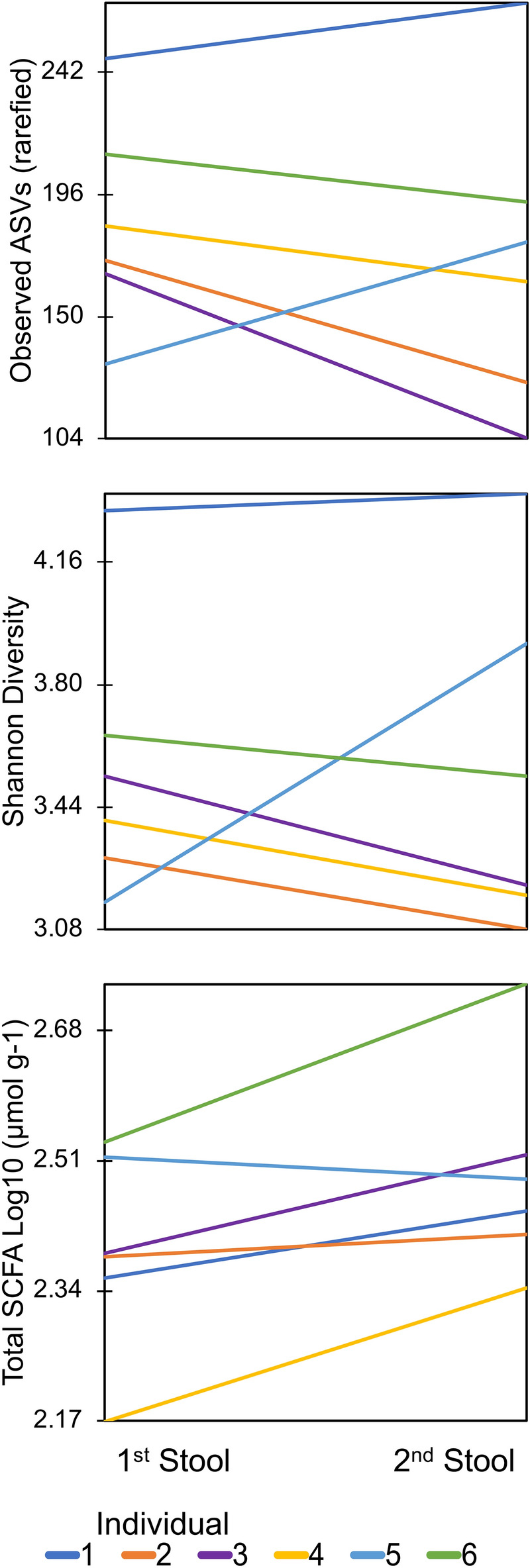
Bacteriome diversity estimates and short chain fatty acid concentration for two consecutive stool collections. Time between stool collections was 8, 2, 4, 10, 4, and 25 h rounded to the nearest whole hour for individuals 1–6 respectively.

All six participants collected two consecutive bowel movements within a 25-h period, with five of the six individuals producing two bowel movements within 10 h. The total SCFA concentration (p = 0.04) and acetic acid concentration (p = 0.03) were significantly higher in the second stool sample compared to the first using a paired t-test. While not significant, butyric acid (p = 0.21) was also higher in the second stool for four of six individuals, while bacterial richness (p = 0.45) and diversity estimates (p = 0.95) were lower in the second stool collection for four of six individuals (Fig. 6). Four ASVs with significant differential abundance in the first stool compared to the second were identified using DESeq2. ASV 137 (Acidaminococcaceae), and 191 (Lachnospiraceae), were enriched in the second stool, while ASV 104 (Dialisteraceae), and ASV 300 (Muribaculaceae) were reduced in the second stool. Additionally, Lachnospiraceae seemed to show a pattern of increased abundance from the first (8%), to the second (15%) stool. Fungal communities from three individuals which were successfully profiled consecutively did not show any trend between richness and diversity measures.

Stool form according to the Bristol Stool Form Scale (BSFS) was also recorded during sample processing, and most individuals (four of six) did not have uniform stool types collected over the three time points. SCFA concentrations clustered in a PCA according to BSFS, and when the SCFA data was integrated with 16S ASV data using rCCA to maximise correlation, the resulting correlation coefficients also grouped according to stool type (Supplementary Fig. 2).

## Discussion

Analysis of the fecal microbiome is now commonly complemented by an additional analysis of microbial metabolites such as SCFA. To ensure these data can be represented together without the impact of spatial and temporal variability of the fecal material, collection and storage methods for stool samples must be considered. Our results found sporadic detection of low abundance bacterial and fungal species in unhomogenized stool. Further, SCFA concentrations were also shown to vary considerably across a single stool.

The level of variability (CV) in microbial diversity and SCFA concentration across a single stool, was compared per participant to the variability across three separate bowel movements. It was expected that temporal shifts in community structure over three timepoints would be larger than replicate sampling from a single stool. While Shannon diversity was more variable for five of six individuals among whole stool samples, Richness based on Chao1 was more variable along a single stool for four of six individuals. As well, SCFA concentrations were more variable within a single stool than across three separate bowel movements for two individuals. When the bacterial and SCFA data was integrated using rCCA, the intraindividual variability between the aliquots was also evident. Clustering of subsamples from individuals 2, 4, and 5 were very tight, indicating little difference in community structure due to sampling method. Although, samples from subjects 1, 3, and 6 were less tightly clustered, implying community structure changes along the surface of the stool in these individuals that are sensitive to sampling method. This demonstrates that for some individuals, heterogeneity of microorganisms and microbial metabolites in stool may be as great as that observed over the course of 2 days, which will become apparent if samples are collected by sub-sampling a small volume of stool. This is consistent with reports of heterogeneity in mucosal bacteriome^19^, fecal microbiome^13,20^, and metabolite concentrations^8^. As fecal material moves through the colon, the exterior surface is exposed to the mucus layer secreted by epithelial cells. This mucus (which is a niche for commensal microbes) accumulates in fecal material, and has been proposed as a mechanism for the patchy recovery of microbial species along the surface of stool^12^.

While our results show the surface of the stool may have more variability in richness and diversity, the β-diversity of bacterial communities between individuals remained significantly different, indicating that relative compositional differences due to subsampling are less pronounced than differences between individuals. This is consistent with similar work, where β-diversity (weighted UniFrac) was compared across nine stool subsampling locations with no significant differences observed^21^. Fungal communities however did not seem to be structured according to the individual to the same extent as bacterial communities, but was structured in one of two ways: dominated by *Saccharomyces cerevisiae* (≥ 99%), or by hosting a more even abundance of genera including S. cerevisiae, and either *Kazachstania servazzii* and *Cyberlindnera jadinii*, or *Hanseniapora uvarum* and *Torulaspora delbrueckii*. In another study targeting fungi in the gut using the ITS1 region, three main mycobiome types were found: either dominated by *Candida albicans*, or *Saccharomyces cerevisiae*, or multi species type^22^. In the present study, *Candida* spp. were not found at greater than 1% of the total community in any individual. However, *Candida apicola* was also not identified in the fungal mock community which could indicate an unknown technical bias against this group, although presumably not due to primer bias as low abundance was detected and this primer set has been used successfully for other Candida species^23^.

Metabarcoding based microbiome investigations are inherently biased as the technique is limited by its semi-targeted design and the compositionality imposed by the NGS technology it uses. This facts is well known in the research community and thus it is encouraged to take corrective steps to reduce any distortion of the “true composition” of the microbial community by unintentional preferences in the workflow for some taxa over others^24^. Bias arises at every stage of the microbiome workflow and has recently been recognized as multiplicative through to bioinformatics, although the largest contributors to this bias are upstream steps such as DNA extraction and PCR amplification^24,25^. The mock communities included in this study indicate that both Bacillus sp., and Clostridium sp., who are active members of the human gut microbiome, seemed to be preferentially targeted by amplification and sequencing. On the other hand, the proportion of *Rhodobacter sphaeroides*, and *Helicobacter pylori* were suppressed, and *Cutibacterium acnes* was not detected. However, suppression of *C. acnes* and *R. sphaeroides* is less concerning given they are not members of gastrointestinal community. It is also important to acknowledge that the sequence data presented here does not represent the actual number of DNA molecules recovered from the stool samples; and is limited by the capacity of the sequencing process. Therefore, the number of reads per sample, or read depth may impact the calculation of β-diversity indices by inflating the between sample diversity of samples with fewer reads^26^. Despite the general move in the field towards accepting that microbiome is compositional, the question of compositionality being driven by NGS or microbiome versus the count origin of microbiome data remains a topic of discussion^27^. Other work on the topic of bias and data correction states that the sensitivity of a β-diversity measure to sequencing effort can also be impacted by the thresholds used to remove rare species^28^, the data normalization approach, and the presence of samples with fewer than approximately 1000 sequences^29^. In this work the widely used Bray–Curtis dissimilarity index was used as a distance measure to illustrate community differences between subjects and collection methods; this enabled us to directly compare our results with prior studies addressing the topic of sample collection. However, this distance measure may not always be the most reliable approach for compositional data with the characteristics previously described^26^.

The microbiome is often scrutinized for small community changes in association with host-related biological factors such as diet or disease. These changes in microbial signatures are often detected in less abundant taxa, or only within particular groups of bacteria and can vary among individuals. Most bacterial ASVs with large differential abundance were found to be enriched in whole stool compared to surface aliquots, and all but one Alphaproteobacteria were classified as Clostridia. The Internal regions of stool have previously been shown to harbor significantly higher abundances of Firmicutes and *Bifidobacteria* spp. compared to the external surface^13^. In this study, the external surface of stool was likely targeted by surface aliquot collections, rather than the internal regions of stool, and if the internal regions of stool harbor larger abundances of Firmicutes, this might explain some of the differences seen between the surface aliquots and the homogenized whole stool. On the other hand, half of all fungi with large differential abundance were found to be reduced in whole stool compared to the surface aliquots; and of these all but one Dothideomycetes were classified as Saccharomycetes, indicating Saccharomycetes may be a mucosal associated fungus in the gut.

The long-term view of the healthy human gut microbiome seems to show a dynamic community which retains prolonged stability, but is punctuated by periods of disturbance^30,31^ On a shorter timescale, diet has been shown to cause fluctuations in microbial species^32^, as well as SCFA concentrations^33^. Furthermore, the microbiome shift caused by daily food choices is highly personal, meaning the same food will elicit a unique response in each individual^34^. What microbiome shifts may look like across consecutive stools has not been previously explored. While only a small proportion of women defecate more than once a day, defecation frequency is known to be higher in men^35^, and positively associated with vigorous physical activity, as well as plant based or high fiber diets^36^. Therefore, the time of day that samples are collected may need to be indicated in sample collection protocols provided to participants. In this study, all women claimed to regularly defecate more than once a day, and the second stool of the day (collected on average 8 h after) had significantly higher total SCFA concentrations, which seemed to be driven by significantly higher concentrations of acetic acid. The second stool also tended to have higher butyric acid concentrations, lower bacterial richness and lower Shannon diversity index compared to the first stool, although these differences were not significant. Similarly, a recent study assessing the microbiome and SCFA concentrations at a single timepoint in 441 adults found that lower bacterial diversity was associated with higher SCFA concentrations^37^. It has been proposed that the gut microbiome has a certain level of volatility which may increase during times of stress^38^, and the level of temporal variance between the two constitutive stools may indicate a normal level of volatility in the microbiome of each individual. Another interesting observation was the similar trend in increase in butyrate producing Lachnospiraceae, and increased butyrate concentrations in the second bowel movement. The association between bacteria and SCFA concentration seen in this study also supports the idea that bacterial metabolites are linked to the circadian clock^39^. Each participant collected the first bowel movement of the day in the morning, followed by the very next bowel movement; and as each woman claimed to regularly defecate at least twice per day, the natural volatility of the microbiome that seems to be linked to the circadian clock demonstrates why time of stool sample collection may be particularly important in individuals who defecate more than once per day.

Decreasing bacterial richness has also been found to correlate with decreasing stool firmness, or a higher Bristol Stool form value, based on fecal samples from 53 women^40^. As well, the BSFS has also been shown to be a good predictor of whole-gut transit time, with high stool form values correlating to longer transit times^35,41,42^. A more recent study also found when stool form had a Bristol Stool value of less than three it was correlated with greater transit times, indicating that stool form can help predict whole-gut or colonic transit times^43^. While this study had a small sample size, it was interesting to note that both SCFA and bacterial phylogenetic diversity grouped according to stool form, and when these data were integrated through rCCA this trend was also observed. The link between transit time, microbial composition and SCFA concentration has been examined in an in-vitro system (Environmental Control System for Intestinal Microbiota). Here, it was shown that reducing the transit time from 48 to 96 h caused a significant decrease in Shannon diversity, as well as an increase in total SCFA concentration^44^, as demonstrated in our study. Quantitative shifts in metabolic analysis between retention times also indicated a metabolic shift in the microbial communities. If microbial diversity and SCFA concentration are linked to transit time, and potentially to stool form as discussed above, assessing stool form at the point of sample processing could be a simple way to add valuable information to downstream multivariate analysis, and help explain sample clustering. Further, to reduce within-day variability that could potentially distort a long-term study, participants could be instructed to collect at a similar time, such as the first bowel movement of the day.

Directly freezing stool samples with no additional solution is considered the gold standard method for storing stool, while Norgen and in OMNIgene·Gut tubes offer a convenient method of collecting fecal material from remote participants. Regardless of collection method, all whole stool samples were dominated by Bacteroidaceae, but the second most abundant family Ruminococcaceae were significantly expanded in samples collected with both the O and N methods compared the F method, indicating that the two preservation methods may impact fecal microbiomes in a similar way. As expected, bacterial β-diversity was mostly driven by inter-individual differences, and this is consistent with previous work where OMNIgene·Gut kits were compared to immediately frozen stool samples^45^. However, unlike Wang^45^ where no significant differences in bacterial β-diversity between these two methods was observed, our data shows significant differences between the three collection methods. The most obvious difference was between the directly frozen samples compared to either of the two other preservation methods (collected at room temperature), and this was observed consistently in all three bowel movements per participant.

Two additional studies have also compared fecal bacterial communities collected using OMNIgene·Gut kits which were frozen prior to processing with samples which were immediately frozen. One study found storage methods, contributed to the significant differences between samples based on Bray–Curtis dissimilarity measure, and that those collected in OMNIgene·Gut kits had a significant increase in Proteobacteria^46^; while another study found that samples stored in OMNIgene·Gut tubes resulted in microbiome profiles with decreased Actinobacteria and increased Lentisphaerae compared to those that were frozen without stabilization^16,45^. Within our study, the preservation tubes were kept at room temperature—in accordance with manufacturer’s instructions—and at the phyla level Actinobacteria were also reduced in fecal samples collected with both the O and N methods. It is more likely then, that the reduction in Actinobacteria is a result of storing in a preservation liquid, rather than the storage temperature.

Stool sample collection methods must not sacrifice sample “viability” for convenience, therefore, where possible we recommend collecting stool in bulk and freezing immediately. As well, during sample processing technicians should take note of the stool form according to BSFS, and homogenize the entire sample prior to subsampling for analysis. This method eliminates any subsampling bias due to heterogenous distribution of microbes in stool, and provides enough material for multiple assays. Additionally, because this method is less hands-on for participants, it may increase compliance if multiple collections are required. For studies where it is not possible to store a large quantity of bulk stool or where frozen transportation of stool is not viable, commercial preservation tubes may be an attractive alternative. In this circumstance it is recommended to only use a single tube type and insure a standard protocol. Furthermore, if OMNIgene·Gut or Norgen collection kits are used, researchers should be cautious in interpreting the reduced abundance of Firmicutes and Actinobacteria. Lastly, collection protocols should consider that some individuals can regularly have more than one bowel movement per day, and those participants should be instructed, where practical, to collect stool at a similar time.

## Methods

### Study design

**Figure 7:**
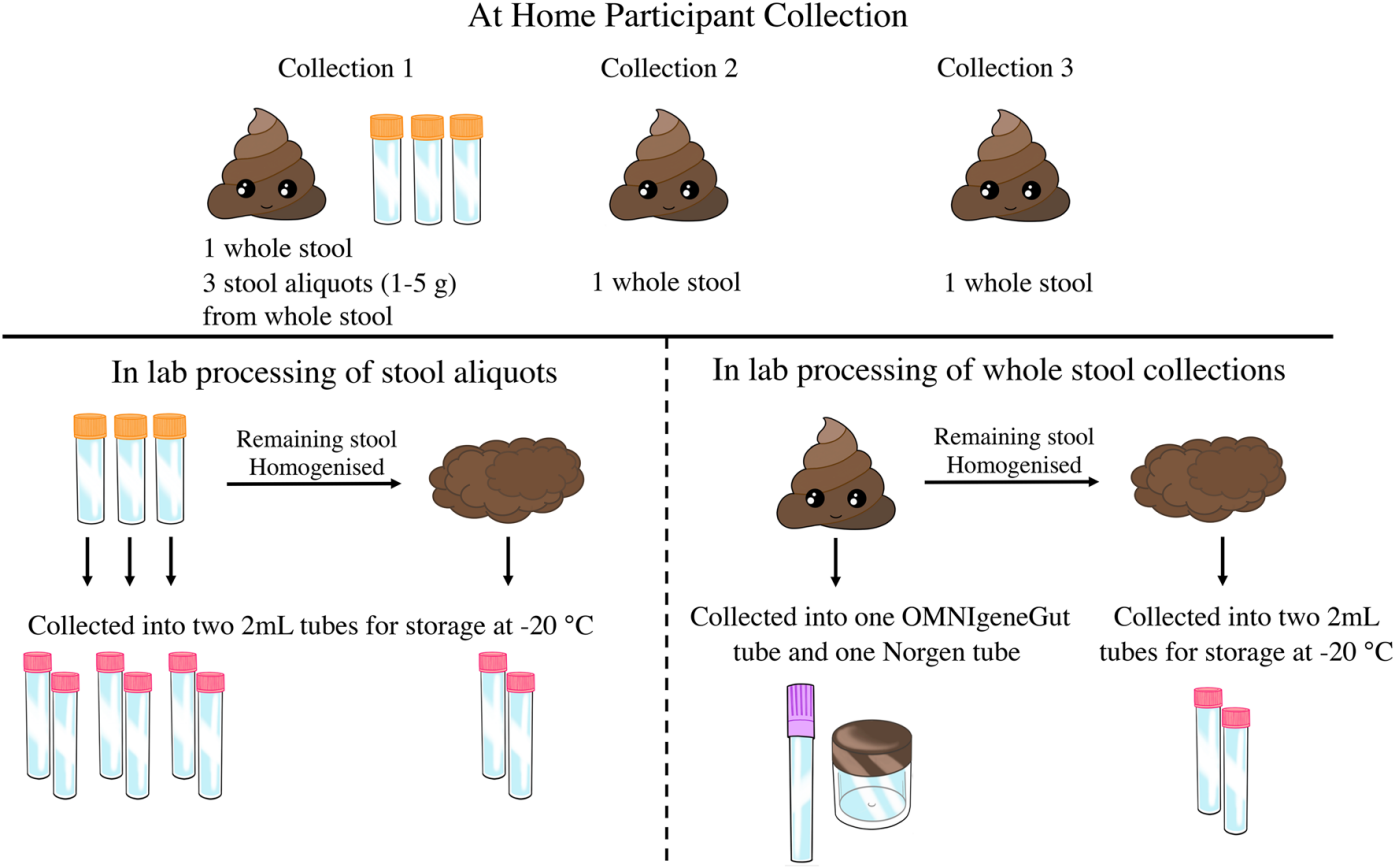
Sample collection by participants at home (top panel), and subsequent sample processing in laboratory (bottom panel). The first bowel movement of the day was obtained for collection 1 and 2, and the very next bowel movement after collection 2 was obtained for collection 3.

Six healthy female volunteers, aged 25–40 years, who had not taken antibiotics in the last 3 months or probiotics in the last month prior to recruitment into this study provided fecal samples with written informed consent. The study protocol was approved by the Human Research Ethics Committee (HRE2018-0791) from Curtin University, Western Australia, and methods were performed in accordance with the relevant guidelines and regulations. Each participant collected three fecal samples at two time points using the provided fecal sample collection kit. All stools were collected at the participants home and frozen immediately (− 20 °C) in a portable freezer. Collection at the first time point (collection 1) required collecting one complete bowel movement, and from this stool collecting three small aliquots in the provided collection tubes. At the second time point, two consecutive bowel movements were collected individually (collection 2 and collection 3), with collection 2 preceding collection 3 (Fig. 7). Once the collection was complete, the freezer was transported to Curtin University and the stool was transferred to a − 80 °C freezer upon arrival.

### Sample preparation

All stool samples were thawed at 4 °C, and transferred on ice to a EuroClone Biological safety cabinet to limit potential contamination. To assess variability between aliquots collected at home, each of the small aliquots were individually homogenized for 30 s with a sterile plastic scoop, and stool (0.25 g) was collected into separate tubes for each of two downstream analyses (metabarcoding, and SCFA quantification). The remaining stool from the initial three aliquots was combined and manually homogenized together for 30 s with a sterile plastic scoop, and collected again for two separate analyses. All samples were immediately frozen to − 80 °C. Prior to preparation, whole stool samples were ranked on the Bristol Stool form chart. To assess collection methods, from each unhomogenized stool, feces were collected into one OMNIgene·Gut tube (DNA Genotec) (collection method O) and one Stool Nucleic Acid Collection and preservation Tube (NORGEN) (collection method N), and were stored at room temperature for 12 days. The remaining stool from each sample was individually homogenized while within the plastic collection bag for 1 min, and then subsequent aliquots of stool (0.25 g) were collected for each of the three analyses and immediately frozen to − 80 °C (collection method F).

### Short chain fatty acid quantification

Homogenized fecal material (0.50 ± 0.05 g) was transported on dry ice to the Science Analytical Facility at Edith Cowan University, Western Australia. Here SCFA were extracted and quantified as previously described^47^. Briefly, an aqueous stock solution of standards containing acetic, propionic, iso-butyric, butyric, iso-valeric, valeric, and hexanoic acids was diluted to four levels, and used for analysis. SCFA were extracted using a solution of hydrochloric acid, methanol, ultrapure water, and 2-ethyl butyric acid which was used as an internal standard. The mixture was vortexed for 1 min, and then incubated at 4 °C for 1 h, and then vortexed a second time for 1 min. Finally, the solution was Centrifuge at 12000 rpm at 4 °C for 20 min, and the supernatant retained and stored at 4 °C for no more than 48 h prior to analysis on Thermo Scientific GC–MS (ISQ) using a Thermo Scientific TG-Wax column (30 m × 0.25 mm × 0.25 µm), and a seven-point calibration. A sample volume of 1.0 µL was injected with an inlet temperature of 220 °C, using Helium carrier gas (1.0 mL/min). The total run time was 18 min.

### Fecal DNA extraction

Immediately prior to DNA extraction, frozen stool samples were thawed on ice, and stool samples stored in preservation tubes were shaken by hand for 10 s. DNA was extracted by using QIAamp Power Fecal DNA kit (QIAGEN, Hilden, Germany) using QIAamp Power Fecal DNA IRT protocol for QIAcube (QIAGEN), as well as OMNIgene·Gut microbial DNA purification protocol using QIAGEN QIAamp PowerFecal DNA kit, both according to the manufacturer’s instructions with one modification at step 3 of the IRT protocol, tubes were vortexed for 20 s to incorporate beads and stool prior to heating. Extraction controls were also processed following the same protocol as frozen stool samples.

### Bacterial and fungal library preparation and sequencing

The V4 region of bacterial DNA and mock communities (ATCC MSA-1002) were targeted and amplified using 16S primers 515F^48^ and 806R^49^, each with a 6–8 bp unique barcode. The PCR reactions contained of 1 × PCR buffer (Applied Biosystems), 2 mM MgCl_2_ (Applied Biosystems), 0.25 nM dNTP (Bioline), 0.4 mg/mL BSA (Thermo Fisher Scientific) 0.4 μM primer (Integrated DNA Technologies), 0.12 × SYBER, and 2U AmpliTaq Gold™ DNA polymerase (Thermo Fisher Scientific). Reactions contained 2 μL of template DNA which was previously screened and optimized for efficiency by qPCR^50^, and had a final volume of 25 μL. Fungal DNA and mock communities^51^ were amplified using ITS2 primers FSeq and RSeq^23^. PCR reactions were the same as for bacterial amplification except 3 μL of template DNA was added to each reaction.

The reactions for both bacterial and fungal amplicons were performed on StepOnePlus Real-Time PCR system (Applied Biosystems), and under the following conditions for bacterial amplicons: denaturing at 94 °C for 3 min, followed by 30 cycles of 94 °C for 40 s, annealing at 53 °C for 40 s, and extension at 72 °C for 60 s. The cycling program for fungi was as follows: denaturing at 94 °C for 3 min, followed by 35 cycles of 94 °C for 40 s, 55 °C for 40 s, 72 °C for 80 s. Both amplicons underwent a final extension at 72 °C for 10 min. Individual bacterial and fungal libraries were prepared by blending together in equimolar concentrations. Illumina compatible adaptors were ligated to the DNA fragments (Lucigen, Middelton, WI, USA), and the resulting amplicons were size selected using Pippin Prep (Sage Science). The QIAquick PCR purification column clean up kit (Qiagen, Germantown, MD), was used to purify the DNA library before sequencing, which was performed at Curtin University, Western Australia, using the Illumina MiSeq platform and V2 500 cycle kit (Illumina, San Diego, CA, USA) with 2 × 250 bp paired-end read lengths.

### Deconvolution

Unique molecular barcodes were used to demultiplex reads with no mismatches allowed. Cutadapt^52^ was used to remove primers, and the remaining reads were quality filtered, trimmed, and merged using DADA2^53^. Reads with ambiguous bases, or with more than two expected errors were discarded. Amplicon sequence variants (ASVs) were inferred from the reads using the pseudo-pooled method, and merged with a minimum overlap of 60 bp allowing for one mismatch (16S V4), and 30 bp with no mismatches (ITS2). Amplicons were retained at a minimum length of 150, and 251, base pairs for ITS2, and 16S V4, datasets respectively. Chimera errors were also removed with DADA2 using the default method. Classification for 16S sequence variants was performed using the Genome Taxonomy reference database (release 95) formatted for use with DADA2 (https://zenodo.org/record/3951383#.X7Hs49sRVTY), while the UNITE general FASTA release for fungi Version 18.11.2018^54^ was used for ITS2 sequence classification, each with a minimum of 50% bootstrapping. Contamination was removed from all sequences with one run of the function remove.count in microDecon^55^. Any ASVs with unassigned phylum, or with a prevalence less than 1 in 5% of samples were filtered out, as were fungal samples with less than 1000 reads.

### Statistical analysis

Sequence counts were used to determine richness and α-diversity indices (Chao1, Shannon, and Faith’s phylogenetic diversity (PD)) for bacterial microbiomes as applied in the “Phyloseq” package^56^ run in R studio with R version 3.6.3^57^. Correlation between library size and diversity estimate were tested for, and α-diversity measures with significant Pearson correlation (p < 0.01) to reads per sample were rarefied to lowest sample depth prior to calculation for those α-diversity measures (Chao1 and Faith’s PD). β-Diversity was compared between collection methods with PERMANOVA in PRIMER-e v7^58^ and visualized using PCoA using Euclidian distances of center log ratio (CLR) transformed data, as well as Bray–Curtis similarity calculated from 4th root transformed proportions of counts. SCFA concentration data were log_10_ transformed, and normality assumed using the Shapiro–Wilk test prior to paired t-test.

To evaluate differentially abundant taxa between homogenization method (aliquots and whole stool), the effect size estimate as a log2 fold change was calculated in DESeq2 statistical package^59^ with a Benjamini–Hochburg adjustment for multiple testing, and a design controlling for subject. Statistical differences between taxa abundance and community diversity due to collection methods were further tested using ANOVA in MicrobiomeAnalyst^60^ after CLR transformation. Lastly, regularized canonical correlation analysis (integrated to maximize correlation between latent variables) was used to integrate SCFA and bacteriome data, both in MixOmics^61^.

## Supplementary Information

Supplementary Information.

## Data Availability

ASV tables, metadata and sequences reads are available in the figshare repository 10.6084/m9.figshare.13670689.
