# Supplementary material for: Fecal sample collection methods and time of day impact microbiome composition and short chain fatty acid concentrations
Source: Sci Rep. 2021 Jul 7;11:13964. doi: 10.1038/s41598-021-93031-z (PMC8263620; doi:10.1038/s41598-021-93031-z)
Supplement: Supplementary file 1 — Supplementary Information. [file 41598_2021_93031_MOESM1_ESM.doc]

**Fecal sample collection methods and time of day impact microbiome composition and short chain fatty acid concentrations**

Jacquelyn Jones, Stacey Reinke, Alishum Ali, Debra Palmer, Claus T. Christophersen

Supplementary Table 1. Sequences of each bacterial and fungal library. Filtering involved all bioinformatic processing including qualify filtering, merging, and trimming, as well as decontamination and low prevalence filtering of ASVs. Richness and diversity estimates are of a single technical replicate sequenced for each gene region

| **Gene region** | **16S, V4** | | **ITS2** | |
| --- | --- | --- | --- | --- |
| Sample sequences post filtering | 4,303,623 | | 8,416,581 | |
| Average reads per sample | 53,387 | | 150,296 | |
| Minimum read count | 5,387 | | 1,073 | |
| Maximum read count | 243,233 | | 1,431,685 | |
| ASVs post filtering | 904 | | 70 | |
| **Technical replicates for each gene region** | |  |  |  |
| Observed ASVs | 285 | 279 | 37 | 39 |
| Shannon | 3.55 | 3.63 | 0.3 | 2.4 |

| Bacterial Mock community composition | 16S V4 region | | | Fungal Mock community composition | ITS2 region | | |
| --- | --- | --- | --- | --- | --- | --- | --- |
|  | F | G | S |  | F | G | S |
| Acinetobacter baumannii |  |  |  | Alternaria alternata |  |  |  |
| Actinomyces odontolyticus |  |  |  | Aspergillus flavus |  |  |  |
| Bacillus cereus |  |  |  | Candida apicola |  |  |  |
| Bacteroides vulgatus |  |  |  | Chytriomyces hyalinus |  |  |  |
| Bifidobacterium adolescentis |  |  |  | Claviceps purpurea |  |  |  |
| Clostridium beijerinckii |  |  |  | Fusarium graminearum / G. zeae |  |  |  |
| Cutibacterium acnes |  |  |  | Fusarium oxysporum |  |  |  |
| Deinococcus radiodurans |  |  |  | Fusarium verticillioides / G. moniliformis |  |  |  |
| Enterococcus faecalis |  |  |  | Mortierella verticillata |  |  |  |
| Escherichia coli |  |  |  | Naganishia albida / C. albidus |  |  |  |
| Helicobacter pylori |  |  |  | Neosartorya fischeri |  |  |  |
| Lactobacillus gasseri |  |  |  | Penicillium expansum |  |  |  |
| Neisseria meningitidis |  |  |  | Rhizoctonia solani |  |  |  |
| Porphyromonas gingivalis |  |  |  | Rhizomucor miehei |  |  |  |
| Pseudomonas aeruginosa |  |  |  | Rhizophagus irregularis |  |  |  |
| Rhodobacter sphaeroides |  |  |  | Saccharomyces cerevisiae |  |  |  |
| Staphylococcus aureus |  |  |  | Saitoella complicata |  |  |  |
| Staphylococcus epidermidis |  |  |  | Trichoderma reesei |  |  |  |
| Streptococcus agalactiae |  |  |  | Ustilago maydis |  |  |  |
| Streptococcus mutans |  |  |  |  |  |  |  |

Supplementary Figure 1. Occurrence of bacterial species of the mock community successfully identified (blue), incorrect classification but resolved correctly at a higher rank (pink), or not identified (grey). Ranks are family (F), genus (G), and species (S)

Supplementary Table 2. Percent coefficient of variation of SCFA concentrations and bacterial diversity estimates among three aliquots collected from a single stool (aliquots) and from three whole stools collected from separate bowel movements (whole). Bolded values indicate the more variable measure per individual. Chao1 and Faith’s phylogenetic distance was calculated on rarefied counts.

| Individual | 1 | | 2 | | 3 | | 4 | | 5 | | 6 | |
| --- | --- | --- | --- | --- | --- | --- | --- | --- | --- | --- | --- | --- |
| Aliquots |  |  |  |  |  |  |  |  |  |  |  |  |
|  | mean(SD) | CV | mean(SD) | CV | mean(SD) | CV | mean(SD) | CV | mean(SD) | CV | mean(SD) | CV |
| Chao1 | 284(27.6) | 9.7 | 200.3(54.2) | **27** | 220.3(17.3) | 7.8 | 174.4(20.2) | **11.6** | 163.1(40.2) | **24.7** | 197.0(25.9) | **13.2** |
| ACE | 330.4(36.4) | 11 | 261.1(74.9) | **28.7** | 273.4(28.3) | 10.3 | 181.4(29.7) | **16.3** | 182.6(38.3) | **21** | 237.4(13.5) | 5.7 |
| Shannon | 4.3(0) | 1.3 | 3.2(0.2) | **6.5** | 3.7(0) | 0.9 | 3.3(0) | 2.1 | 3.4(0.1) | 4.9 | 3.4(0) | 0.3 |
| Faith’s PD | 26.8(1.2) | 4.4 | 19.9(1.3) | 6.6 | 21.5(2.3) | 10.6 | 16.7(1.2) | **7.7** | 16.7(3.4) | **20.7** | 17.6(0.4) | **2.6** |
| acetic acid | 127.5(34.7) | **27.1** | 113(13) | 11.5 | 143.9(26) | 18.1 | 202.5(12) | 5.9 | 229.6(23.9) | **10.4** | 486.6(183.2) | **37.6** |
| propionic acid | 28.1(4.6) | **16.4** | 19.5(0.8) | 4.2 | 24.8(2.8) | 11.5 | 33(1.1) | 3.5 | 58.2(3.7) | 6.4 | 71.3(12) | 16.8 |
| butyric acid | 28.4(4.2) | **14.9** | 16.5(2.5) | 15.3 | 26.7(3) | 11.2 | 37.4(1.4) | 3.8 | 33.6(2.3) | 7 | 83.8(37.8) | **45.2** |
| valeric acid | 4.7(0.8) | **19** | 2.9(0.1) | 3.5 | 2.2(0.4) | 20 | 3.9(0.2) | 5.9 | 2.3(0.1) | 7.6 | 9.4(3.7) | **39.9** |
| total | 188.8(41.5) | **22** | 152.1(9.7) | 6.4 | 197.8(32) | 16.2 | 276.9(13.9) | 5 | 323.8(27.6) | **8.5** | 651.2(164.2) | **25.2** |
| Whole |  |  |  |  |  |  |  |  |  |  |  |  |
|  | mean(SD) | CV | mean(SD) | CV | mean(SD) | CV | mean(SD) | CV | mean(SD) | CV | mean(SD) | CV |
| Chao1 | 264.3(36.4) | **13.8** | 178(38.2) | 21.5 | 185.2(64.8) | **35** | 197.6(19.7) | 10 | 190.1(40.23) | 21.2 | 244.7(3.1) | 1.3 |
| ACE | 301(49.1) | **16.3** | 232.9(57.2) | 24.5 | 227(93.4) | **41.1** | 232.8(12.9) | 5.5 | 203.3(24.3) | 11.9 | 280.2(17.3) | **6.1** |
| Shannon | 4.2(0) | **2** | 3.1(0.1) | 3.3 | 3.4(0.1) | **5.7** | 3.3(0.1) | **4.4** | 3.5(0.3) | **10.6** | 3.5(0) | **2.4** |
| Faith’s PD | 26.3(2.6) | **10** | 19.3(3.4) | **17.8** | 19.3(3.1) | **16.3** | 17.9(0.9) | 5.2 | 17.3(2) | 11.8 | 19.8(0.4) | 2.2 |
| acetic acid | 178.5(23.5) | 13.1 | 171.7(40.4) | **23.5** | 185.2(56.8) | **30.6** | 144.8(42.3) | **29.2** | 236.7(5.5) | 2.3 | 347.8(103.4) | 29.7 |
| propionic acid | 32.7(0.7) | 2.2 | 22.9(1.2) | **5.5** | 32.1(8.4) | **26.1** | 26.7(9.9) | **37.2** | 52.3(15.3) | **29.3** | 52(16.1) | **31** |
| butyric acid | 33.6(2.2) | 6.7 | 27.3(7.3) | **27** | 32.4(7.1) | **21.9** | 38.5(11) | **28.5** | 33.1(4.9) | **14.8** | 72(30) | 41.7 |
| valeric acid | 5.3(0.3) | 7.1 | 2.5(0.7) | **27.7** | 2.8(0.5) | **20.1** | 3.4(1.2) | **35** | 2.7(0.3) | **11.8** | 6.6(1.3) | 20.8 |
| total | 250.3(25.6) | 10.2 | 224.6(45.6) | **20.3** | 252.7(72.6) | **28.7** | 213.6(63) | **29.5** | 325(18.4) | 5.6 | 478.6(118.4) | 24.7 |

Supplementary Table 3. Taxa identified in significantly different abundance in the O or N methods compared to the F method.

|  |  | F vs N | |  |  | F vs O | |
| --- | --- | --- | --- | --- | --- | --- | --- |
|  |  | Pvalues | FDR |  |  | Pvalues | FDR |
| **Phyla** | |  |  | **Phyla** | |  |  |
|  | Firmicutes | 0.008 | 0.092 |  | Actinobacteriota | 0.013 | 0.160 |
|  | Actinobacteriota | 0.023 | 0.140 |  | Bacteroidota | 0.039 | 0.234 |
| **Class** | |  |  | **Class** | |  |  |
|  | Clostridia | 0.003 | 0.059 |  | Negativicutes | 0.002 | 0.032 |
|  | Lentisphaeria | 0.031 | 0.229 |  | Coriobacteriia | 0.003 | 0.032 |
|  |  |  |  |  | Bacilli | 0.030 | 0.185 |
|  |  |  |  |  | Bacteroidia | 0.039 | 0.185 |
| **Order** | |  |  | **Order** | |  |  |
|  | Lachnospirales | 0.001 | 0.056 |  | Monoglobales | 0.001 | 0.058 |
|  | Oscillospirales | 0.005 | 0.077 |  | Coriobacteriales | 0.003 | 0.068 |
|  | Not_Assigned | 0.006 | 0.077 |  | Lactobacillales | 0.010 | 0.131 |
|  | Rhizobiales | 0.017 | 0.174 |  | Bacteroidales | 0.042 | 0.381 |
|  | Victivallales | 0.031 | 0.250 |  |  |  |  |
|  | Monoglobales | 0.041 | 0.254 |  |  |  |  |
| **Family** | |  |  | **Family** | |  |  |
|  | Ruminococcaceae | 0.001 | 0.041 |  | Ruminococcaceae | 0.000 | 0.003 |
|  | Lachnospiraceae | 0.001 | 0.041 |  | Eggerthellaceae | 0.000 | 0.003 |
|  | Actinomycetaceae | 0.002 | 0.047 |  | UBA1381 | 0.001 | 0.035 |
|  | Eggerthellaceae | 0.008 | 0.138 |  | Rikenellaceae | 0.004 | 0.080 |
|  | Oscillospiraceae | 0.010 | 0.151 |  | Actinomycetaceae | 0.011 | 0.161 |
|  | Rhizobiaceae | 0.017 | 0.186 |  | Streptococcaceae | 0.015 | 0.184 |
|  | Not_Assigned | 0.018 | 0.186 |  | Acutalibacteraceae | 0.018 | 0.184 |
|  | Victivallaceae | 0.031 | 0.281 |  | Veillonellaceae | 0.025 | 0.221 |
|  | UBA1381 | 0.041 | 0.327 |  | Erysipelatoclostridiaceae | 0.037 | 0.297 |

Supplementary Figure 2. Bacterial communities and metabolites group according to Stool form. SCFA and 16S ASV from three matching whole stool samples were combined using rCCA in the R package MixOmics, and plotted as correlation coefficients.
